# Synthesis and Optical Properties of In_2_S_3_-Hosted Colloidal Zn–Cu–In–S Nanoplatelets

**DOI:** 10.1021/acsomega.1c02180

**Published:** 2021-07-16

**Authors:** Ze Yuan, Lanlan Yang, Dongni Han, Guorong Sun, Chenyu Zhu, Yao Wang, Qiao Wang, Mikhail Artemyev, Jianguo Tang

**Affiliations:** † Institute of Hybrid Materials, National Center of International Joint Research for Hybrid Materials Technology, National Base of International Science & Technology Cooperation on Hybrid Materials, Qingdao University, 308 Ningxia Road, Qingdao 266071, People’s Republic of China; ‡ Research Institute for Physical Chemical Problems of the Belarusian State University, Minsk 220006, Belarus

## Abstract

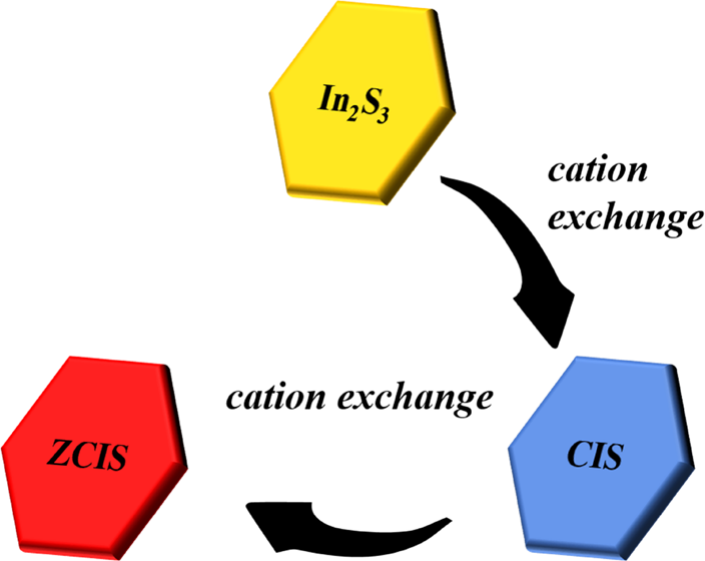

High-efficiency photoluminescence quaternary hexagon Zn–Cu–In–S (ZCIS) nanoplatelets (NPls) have been synthesized by a two-step cation exchange method, which starts with the In_2_S_3_ NPls followed by the addition of Cu and Zn. It is the first time that In_2_S_3_ NPls are used as templates to synthesize ZCIS NPls. In this paper, the reaction temperature of In_2_S_3_ is essential for the formation of NPls. The photoluminescence wavelength of NPls can be tuned by adjusting the temperature of Cu addition. To enhance the stability of the resulting NPls and to improve their optical properties, we introduced Zn^2+^ and obtained ZCIS NPls by cation exchange on the surface. It is worth noting that the obtained ZCIS NPls show a shorter fluorescence lifetime than other ternary copper sulfide-based NPls. This work provides a new way to synthesize high-efficiency, nontoxic, and no byproduct ZCIS NPls.

## Introduction

Recently, two-dimensional (2D) colloidal semiconductor nanoplatelets (NPls) have attracted scientific and practical interest due to their unique anisotropic optical and electronic properties. In the past few years, the synthesis of NPls in solution has been developed in detail but focused mostly on binary metal chalcogenide compounds, including CdX (X = S, Se, Te),^1−4^ PbS,^5−7^ PbSe,^4,7^ Cu_2–*x*_S,^8−10^ and Cu_2–*x*_Se.^11−14^ Because such NPls either contain heavy metals (Cd, Pb) or are based on indirect gap semiconductors (Cu_2–*x*_S), this limits their practical applications in biomedicine or optoelectronics.

In the past decade, the focus on the synthesis and utilization of colloidal semiconductor nanocrystals (NCs) has been shifted toward more “greener” ternary CuInS_2_, CuInSe_2_, or AgInS_2_ compounds.^15−18^ Among others, CuInS_2_ (CIS) has been the most popular semiconductor for light-absorbing or light-emitting applications.^17−21^ CIS has a direct bulk band gap of 1.45 eV^22−24^ and the Bohr radius of exciton of 4.1 nm. Due to the quantum confinement effect in (quasi) spherical CIS NCs, the spectral range of photoluminescence (PL) spans between 500 and 1000 nm,^17,25−29^ making CIS NCs useful in broad practical applications. Pure CIS NCs usually exhibit a relatively low PL quantum yield due to the presence of surface defects. The standard approach to increase the quantum yield in CIS NCs involves the partial cation exchange with Zn, resulting in the “core–gradient shell” ZCIS structure with the core enriched with Cu and In and the shell with Zn. Unlike (quasi-)spherical NCs, CIS(Se) NPls were not widely established and rarely published.^6,30,31^ Earlier, Lox et al. used CuS nanodiscs as a template to obtain ZCIS NPls by the two-step cation exchange.^32^ However, ZCIS NPls obtained by such a method produce byproducts during the first cation exchange with Cu, which affects the PL characteristics of ZCIS NPls.

Contrary to previously published procedures, in this paper, we propose to use In_2_S_3_ NPls as a template to obtain ZCIS NPls through a two-step cation exchange reaction: partial replacement of In^3+^ ions with Cu^+^ followed by addition of Zn ions. Earlier, we successfully used this approach to synthesize highly luminescence ZCIS QDs.^33^ As-synthesized ZCIS NPls preserve the hexagonal morphology of their In_2_S_3_ predecessor while demonstrating an intense PL signal in the red part of the visible spectrum presumably due to formation of a core–shell structure.

## Results and Discussion

**Figure 1:**
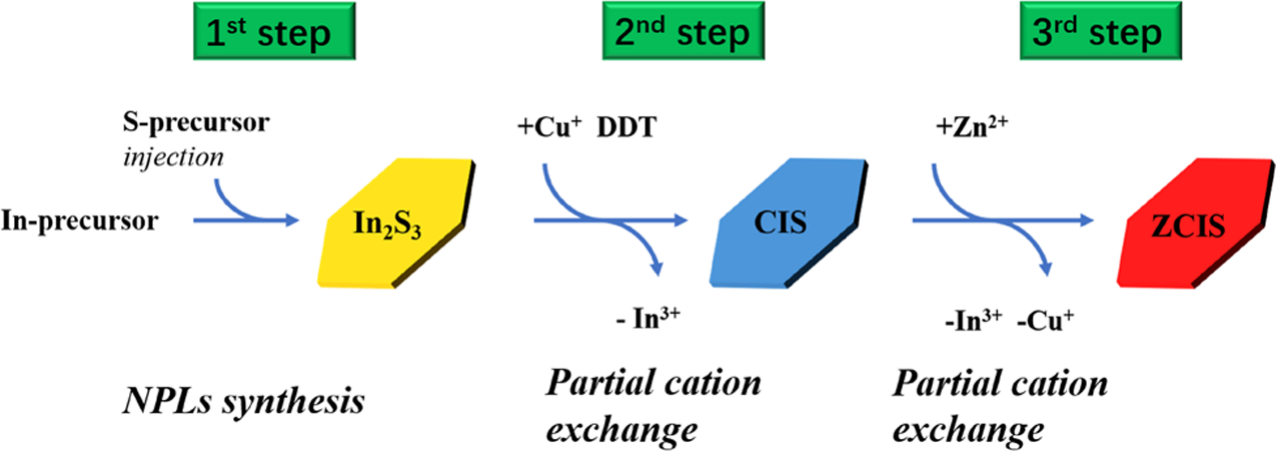
Scheme of the synthesis of ZCIS NPls including preparation of In_2_S_3_ NPls followed by two-step cation exchange with Cu and then Zn ions.

Figure 1 shows the step-by-step scheme of the synthesis of ZCIS NPls.

**Figure 2:**
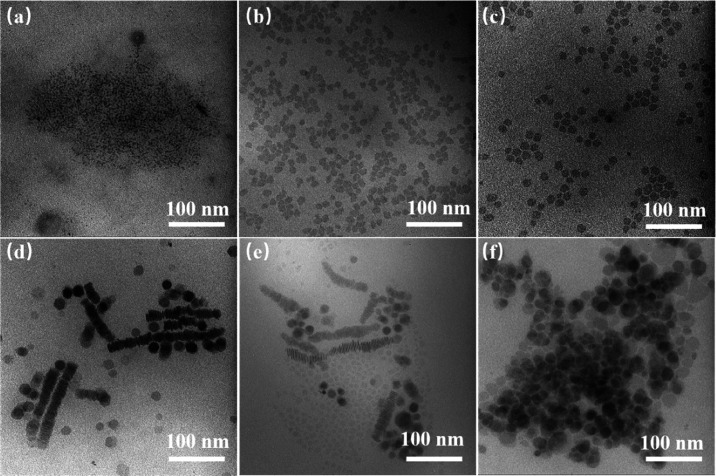
TEM images of In_2_S_3_ NPls formed at different reaction temperatures: 140 °C (a), 160 °C (b), 180 °C (c), 200 °C (d), 220 °C (e), and 240 °C (f).

The starting point of our work was to establish the reaction conditions for the formation of In_2_S_3_ NPls by the reaction between In(III) chloride and sulfur in ODE. To ensure that sulfur will not react with other cations to form separate quantum dots, double quantity of InCl_3_·4H_2_O was used to exhaust sulfur completely. Figure 2 demonstrates representative TEM images of In_2_S_3_ NCs formed at different reaction temperatures. When the temperature is below 160 °C, In_2_S_3_ exists in the form of quasi-spherical QDs. When the temperature increases to 160 °C, the NPls begin to form. With increased reaction temperature, the morphology of the NPls gets a regular hexagonal shape and a larger lateral size (Figure S1 of the Supporting Information). Interestingly, when the reaction temperature is maintained around 200–220 °C, the NPls tend to stack on the TEM grid, which may point to their high homogeneity in the size and shape and atomically flat surface as seen before in CdSe NPls.^34,35^ As the temperature reaches 240 °C, the NPls lose their hexagonal shape, getting broken edges and broadened lateral size distribution. The stacks also disappear as a result of nonideality in the NPl morphology. Therefore, Figure 2 shows that to obtain CIS NPls with the regular morphology, the optimum injection temperature for the Cu precursor lies between 160 and 200 °C.

**Figure 3:**
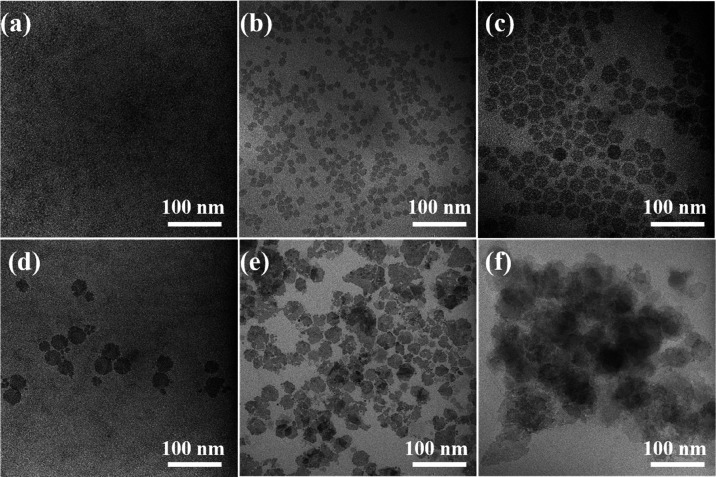
TEM images of CIS NPL obtained by injecting the Cu precursor at different temperatures: 140 °C (a), 160 °C (b), 180 °C (c), 200 °C (d), 220 °C (e), and 240 °C (f).

In the second step, after the formation of In_2_S_3_ NPls, a Cu precursor is injected into the reaction mixture in the form of CuI dissolved in DDT. DDT acts as a solvent and reduces the reactivity of Cu ions at an appropriate temperature, thereby increasing the control over the exchange reaction and avoiding the generation of separate Cu*_x_*S NCs. Since the reaction temperature at the first stage governs the morphology of In_2_S_3_ NPls, we introduced the Cu precursor at a different reaction temperature to explore how it affects the formation of CIS NPls (Figure 3).

When the temperature is as low as 140 °C, the NPls do not form, and the final product consists of quasi-spherical NCs. On the other side, when the temperature exceeds 200 °C, the NPls lose their homogeneity and possess a somewhat irregular shape with holes and broken edges. Therefore, 180 °C is considered the optimum temperature for cation exchange with Cu since it produces NPls with the most regular and less defective shape.

**Figure 4:**
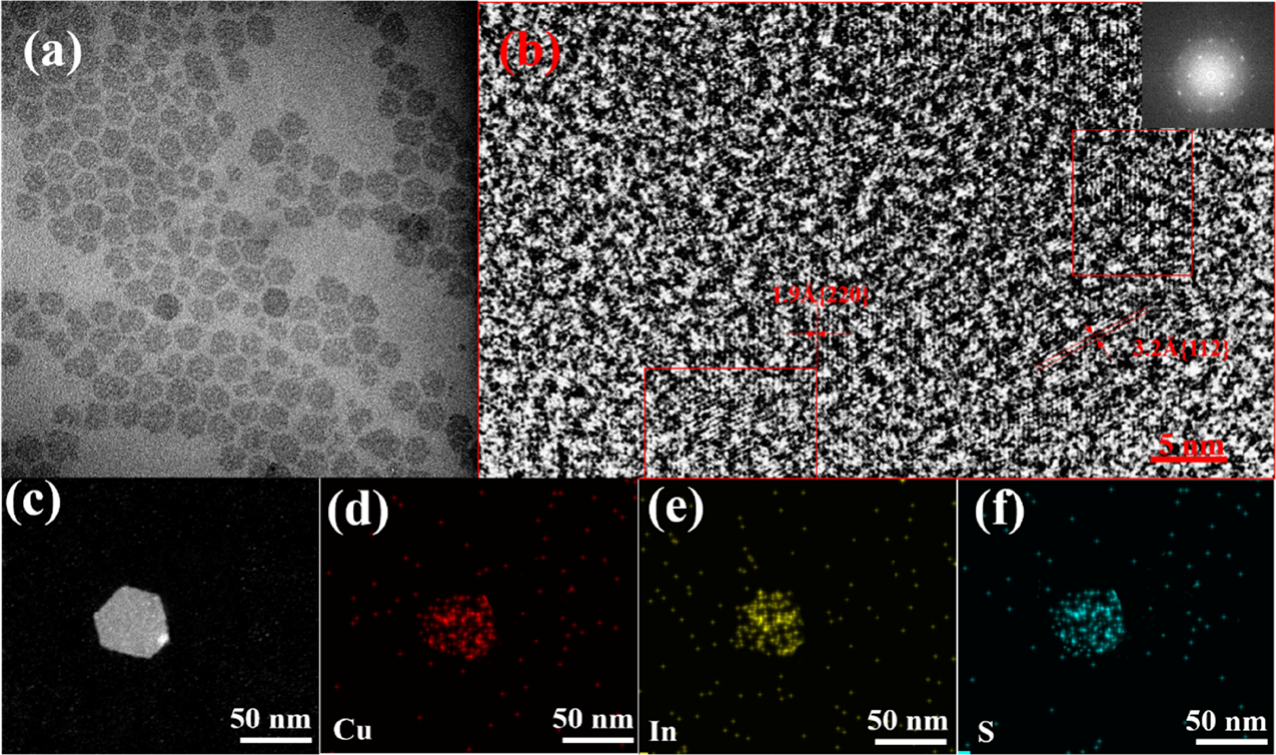
TEM (a) and HRTEM (b) images of CIS NPls and Fourier transform; (c) HAADF-STEM image of CIS NPls; and STEM-EDS elemental maps showing the distribution of Cu (d), In (e), and S (f) atoms.

To analyze whether Cu is introduced homogeneously into an In_2_S_3_ matrix, we performed STEM-EDX elemental mapping of NPls obtained by the injection of a Cu precursor at 180 °C. Figure 4 shows that In, Cu, and S are distributed homogeneously over the ensemble of NPls. EDS elemental analysis points to the Cu/In/S = 0.8:1:1.9 atomic ratio close to stoichiometric CuInS_2_ and indicates the absence of byproducts in the ensemble of CIS NPls. HRTEM analysis of the crystalline structure of individual CIS NPls in Figure 4b shows that it has a single-crystal hexagonal structure with the *d*-spacing of 3.2 Å, corresponding to that of the tetragonal roquesite phase CuInS_2_ (JCPDS No. 38-0777). Compared to the initial In_2_S_3_ NPls, the size of CIS NPl nanocrystals increases by 20% (see Figures S2 and S3 in the Supporting Information). Hence, Cu in CIS NPls is introduced via In atom substitution rather than by a reaction between Cu and S precursors.

**Figure 5:**
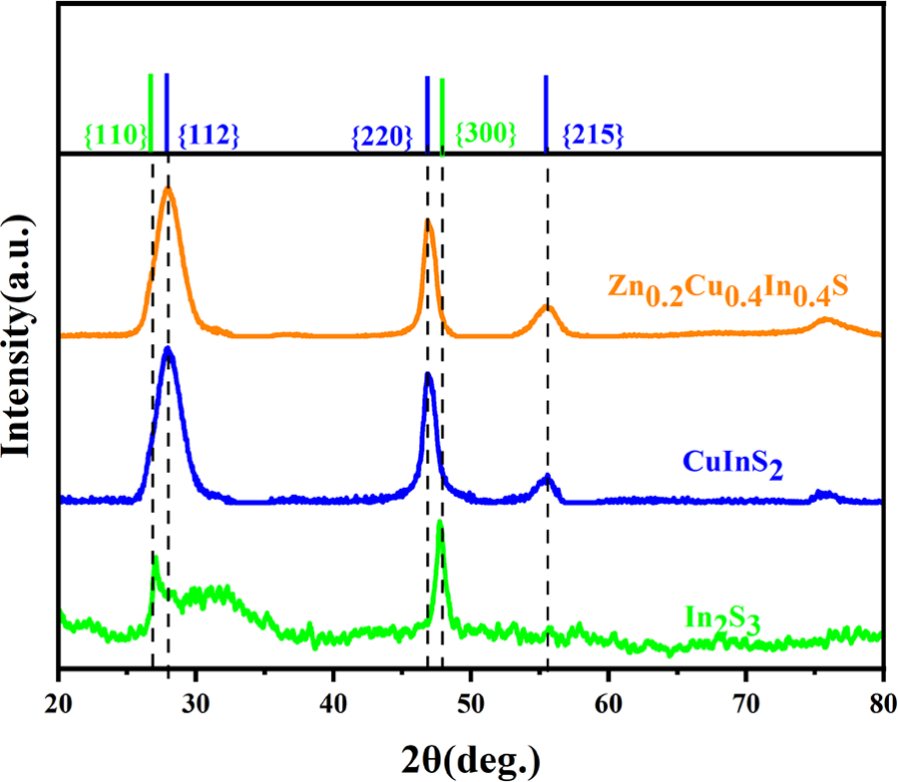
XRD patterns of powdered In_2_S_3_, CIS, and CZIS NPls. Corresponding reference data are indicated as color bars at the top of graphics for bulk In_2_S_3_ (green), CuInS_2_ (blue), and Zn_0.2_Cu_0.4_In_0.4_S (orange).

XRD data in Figure 5 demonstrate that the starting In_2_S_3_ NPls are in the hexagonal phase. The reflexes from {110} and {300} crystalline planes at 27.156 and 47.995°, respectively, are shifted to the shorter angles, which along with the decreased intensity of the {300} reflex confirm the 2D structure of In_2_S_3_ NPls with weak distortion along the {300} direction. After the incorporation of Cu ions, the crystal structure changed to a tetragonal roquesite phase.

Using the Debye–Scherrer formula, we estimated the crystallite size of CIS NPls (∼39 nm over the ⟨112⟩ direction), close to the lateral dimension determined from TEM (see Table 1), which indicates that CIS NPls preserve a monocrystalline structure after the cation exchange.

**Table 1:** Average Lateral Size (According to TEM), Thickness, and the Cu/In/S/Zn Atomic Ratio of In_2_S_3_, CIS, and ZCIS NPls

NPl sample	lateral dimension (nm)	thickness (nm)	ratio of the element
In_2_S_3_ NPls	25 ± 5	2.5 ± 0.5	In/S = 0.7:1
CIS NPls	35 ± 5	3.5 ± 0.3	Cu/In/S = 0.42:0.52:1
ZCIS NPls	45 ± 5	4.1 ± 0.3	Zn/Cu/In/S = 0.29:0.38:0.42:1

We conducted the Raman test and added the data to Figure S5 of the Supporting Information. CIS NPls show a broad peak that appears near 352 cm^–1^, while the introduction of Zn shifts this peak to ca. 350 cm^–1^. This peak is characteristic of the tetragonal Cu–In–S phase with a significant copper deficiency.^36,37^ The broad peak between 550 and 700 cm^–1^ can be related to a second-order scattering process. The Raman data show that the introduction of Zn does not change the crystalline structure of CIS, which is consistent with the XRD data.

**Figure 6:**
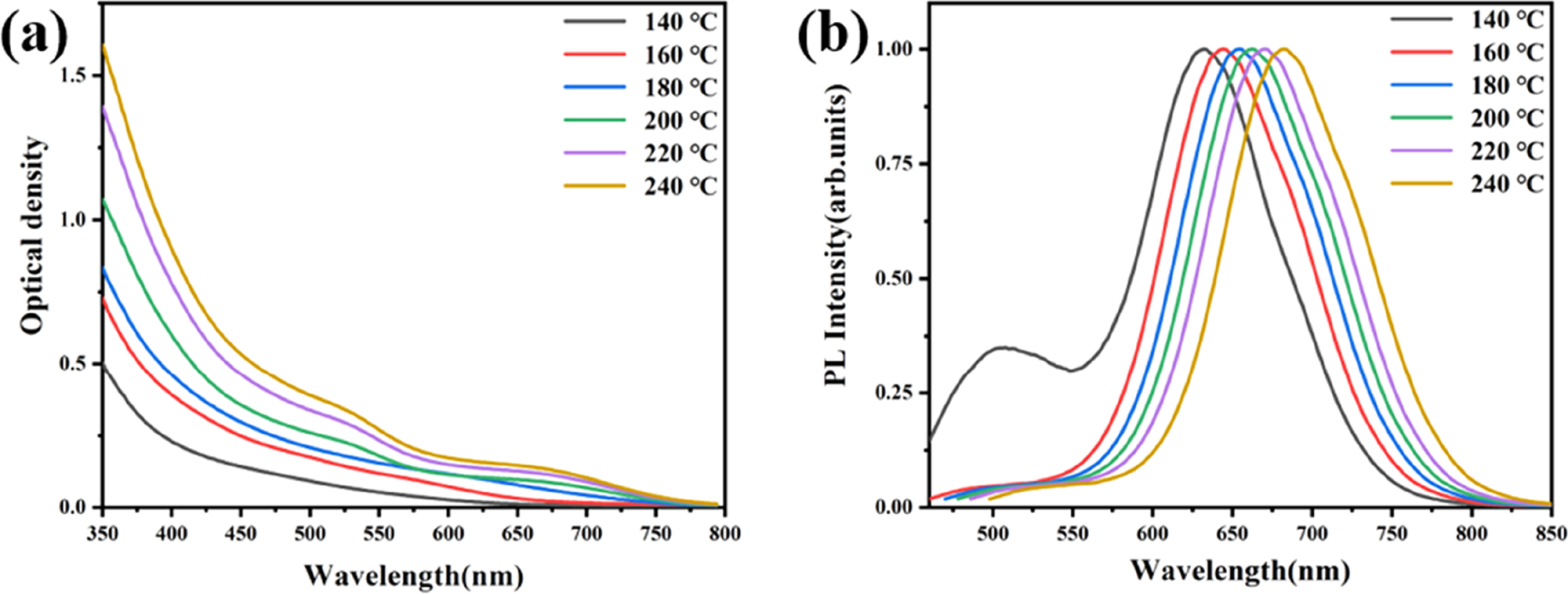
Absorption (a) and PL spectra (b) of colloidal solutions of CIS NPls obtained at different temperatures of Cu precursor injection indicated on the graphs.

Figure 6 shows optical absorption and PL spectra of CIS NPls obtained at different reaction temperatures of Cu precursor injection.

The cation exchange that proceeded at the lowest applied temperature of 140 °C results in the appearance of a well-resolved PL peak around λ ≈ 620 nm, which can be assigned to CIS NPls. In parallel, we see the presence of another, much weaker PL peak around λ ≈ 500 nm probably related to nonreacted In_2_S_3_ or ultrasmall CIS QDs as a product of partial breaking of CIS NPls (Figure 3). An increase in the reaction time results in the long-wavelength shift of the PL band either due to an increase in the Cu content or the NPl thickness (the thicker the NPls, the weaker is the transverse quantum confinement). Below 180 °C, the corresponding absorption spectra are almost featureless with the optical band gap at λ ≈ 600–620 nm (2.06–2.0 eV). Above 200 °C, a broad first excitonic transition appears at λ ≈ 650 nm (1.9 eV), which shifts to λ ≈ 670 nm (1.85 eV) at 240 °C. Another, higher energy excitonic transition appears around λ ≈ 525 nm above 200 °C. The absence of well-resolved peaks in absorption spectra of CIS NPls together with a broad-band emission in the red part of the visible spectrum is similar to spherical CIS QDs, widely studied earlier (see, for example, ref (33) and the references therein). The strong spectral broadening of the optical transitions in CIS QDs (and NPls) relate either to an inhomogeneous size distribution or elemental composition.

Due to surface defects, the PL quantum yield of CIS NPls is relatively low, around 1%. To improve this parameter important for practical applications, we performed a second cation exchange step by adding a Zn precursor to the as-synthesized CIS NPls to form quaternary ZCIS NPls, similar as done earlier with CIS QDs.^33^ The addition of a fourth element (Zn) could cause damage to the NPls due to crystalline lattice distortion. To avoid damaging NPls, Zn was introduced under the mild temperature. To complete the cation exchange, we slowly increased the temperature to 240 °C.

**Figure 7:**
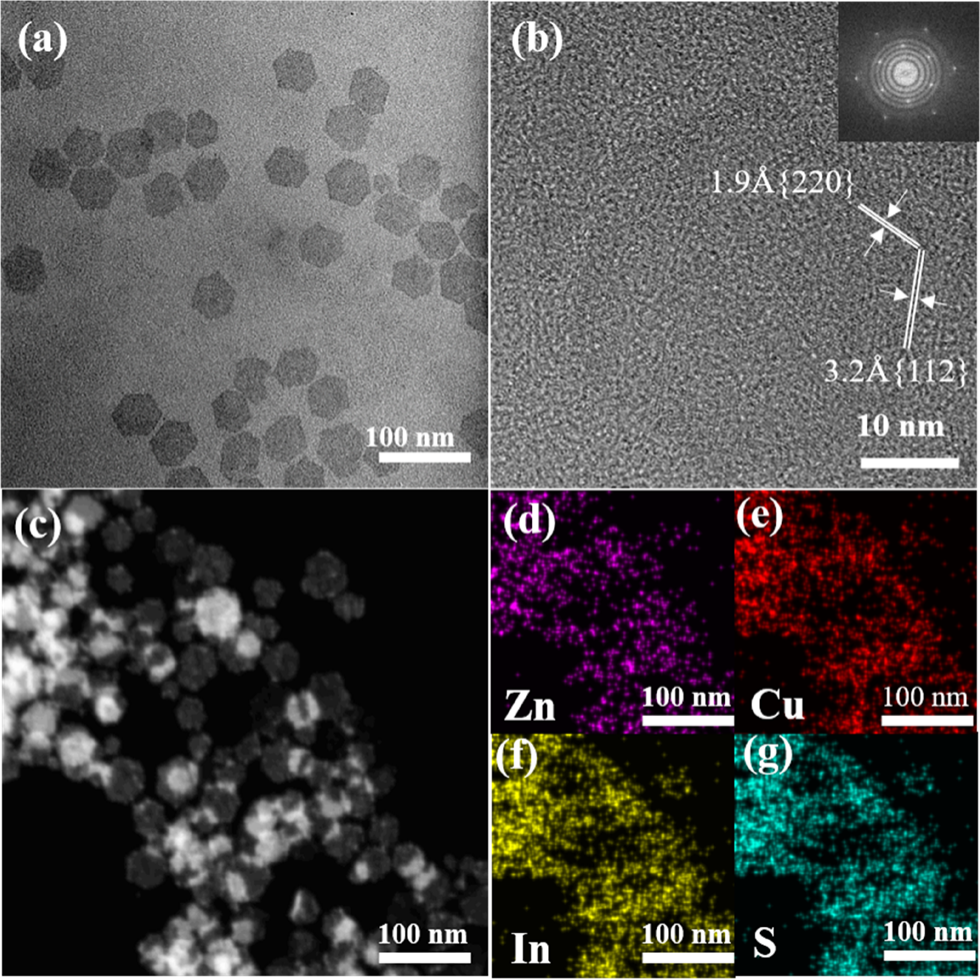
TEM (a) and HRTEM (b) images of ZCIS NPls and Fourier transform; (c) HAADF-STEM image of ZCIS NPls; and corresponding STEM-EDS elemental maps showing the distribution of Zn (d), Cu (e), In (f), and S (g) atoms.

Figure 7 demonstrates that the shape of the obtained ZCIS NPls remains hexagonal, the same as that of CIS NPls. The lateral size of the ZCIS NPls and their thickness increase from 35 ± 5 to 45 ± 5 nm and from 3.5 to 4.1 nm, respectively (Table 1). Acording to the FTIR spectrum presented in Figure S6 of the Supporting Information, ZCIS NPls contain oleylamine surface ligand molecules.

According to the STEM-EDS data, the Zn/Cu/In/S ratio in synthesized ZCIS NPls is 0.29:0.38:0.42:1. The chemical composition of ZCIS NPls is close to ZCIS QDs obtained by a similar protocol.^33^ HRTEM and FT analyses show that the synthesized ZCIS NPls still retain the same crystal structure as CIS NPls. Figure 7b shows characteristic electron diffraction rings corresponding to the {112} and {220} crystal planes. The XRD data in Figure 5 shows that incorporation of Zn into a CIS matrix does not markedly affect the crystalline lattice parameters of NPls.

**Figure 8:**
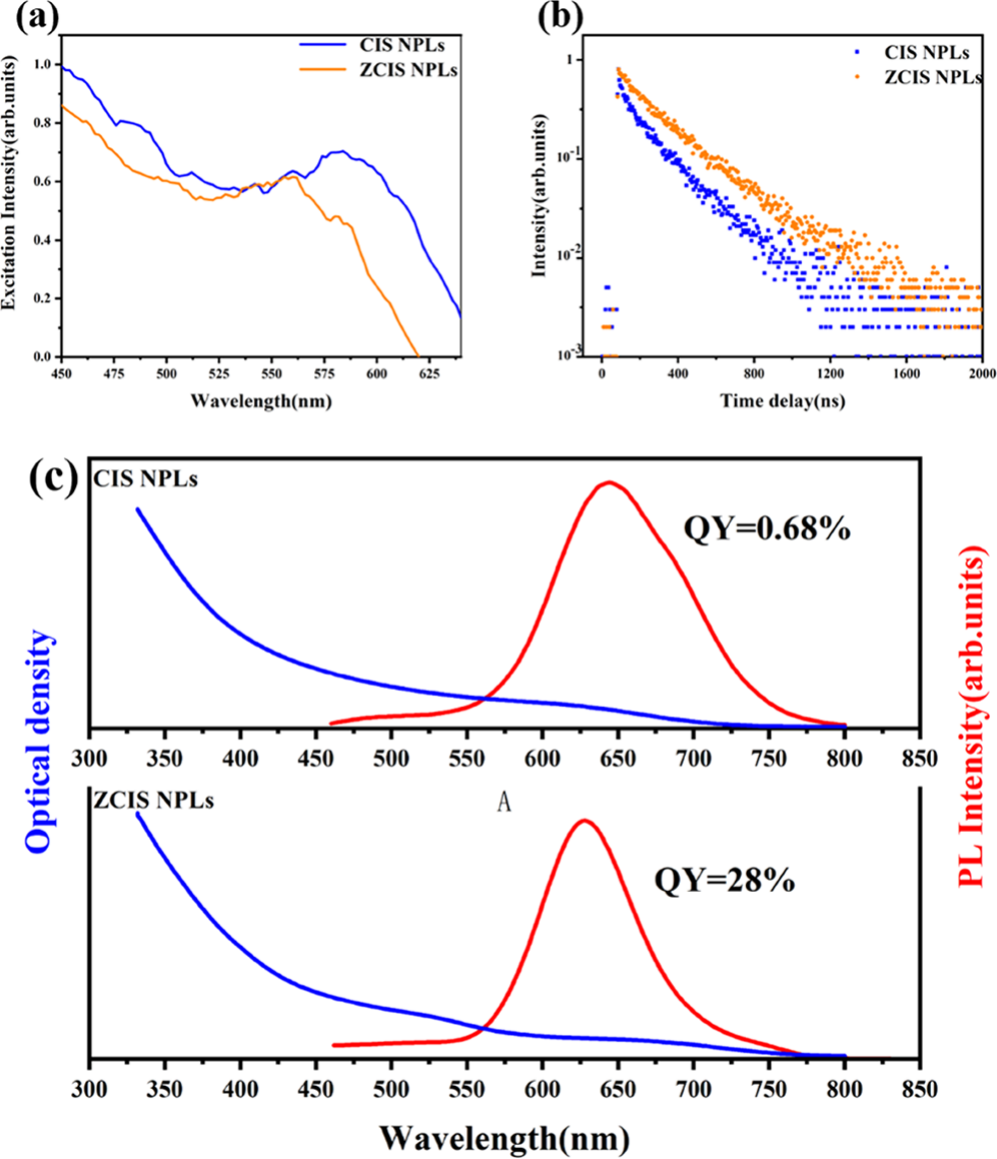
Excitation spectra (a) of CIS NPls (λ_em_ = 650 nm) and ZCIS NPls (λ_em_ = 630 nm); PL decay curves (b) for ZCIS NPls; and optical absorption (blue), PL (red), and PL QY of CIS NPls and ZCIS NPls (c). λ_ex_ = 440 nm.

Figure 8 demonstrates that after the incorporation of Zn into CIS NPls, a broad excitonic absorption band around λ ≈ 650 nm splits into two components: the first shifts to ca. 550 nm, while the second to 720 nm. The PL band follows the short-wavelength trend and shifts from λ = 660 to 630 nm and strongly increases in intensity with the incorporation of Zn. Interestingly, PLE spectra do not correlate with the absorption ones: PLE maximum for CIS NPls shifts to the blue on ca. 70 nm, close to the value of the Stokes shift between PL and PEL peaks. A weak long-wavelength shoulder in the PL spectrum of CIS NPls points to strong inhomogeneity in thickness or chemical composition. The PL quantum yield measured against rhodamine 6G was below 1% in CIS NPls and increases to 28% in ZCIS NPls due to eliminating surface traps. Moreover, the addition of Zn^2+^ increased the fluorescence lifetime of NPls from 197 ns for CIS to 243 ns for ZCIS with the monoexponential character of the decay curve for the same reason. This PL decay time, spectral linewidth (ca. 80 nm), and the corresponding Stokes shift around 70 nm lie within the previously established values for ZCIS QDs^30,38^ and should point to the dopant-type emission from Cu^+^ vacancies.^39^ However, the ZCIS absorption spectrum lasts far more into the red part as compared to CIS counterparts. Such a long-wavelength component characterized by a low PL quantum yield can be attributed to a separate Cu-enriched phase, probably QDs formed during partial destruction of CIS NPls during Zn incorporation followed by recrystallization of residuals into compact NCs. We stored as-synthesized ZCIS NPls in a colloidal solution in dark at room temperature for 3 months and then performed the photoluminescence, absorption, and TEM tests. Figure S7a,b demonstrates PL and absorption spectra of ZCIS NPls before and after long-term storage. As we see from this figure, the absorbance and photoluminescence did not change significantly after being stored in the dark environment for 3 months. This indicates that ZCIS NPls have good stability of their intrinsic crystalline structure and morphology and, as a consequence, the optical characteristics. Figure S7c shows the freshly prepared ZCIS NPls and Figure S7d shows the ZCIS NPls stored in the dark for 3 months.

**Figure 9:**
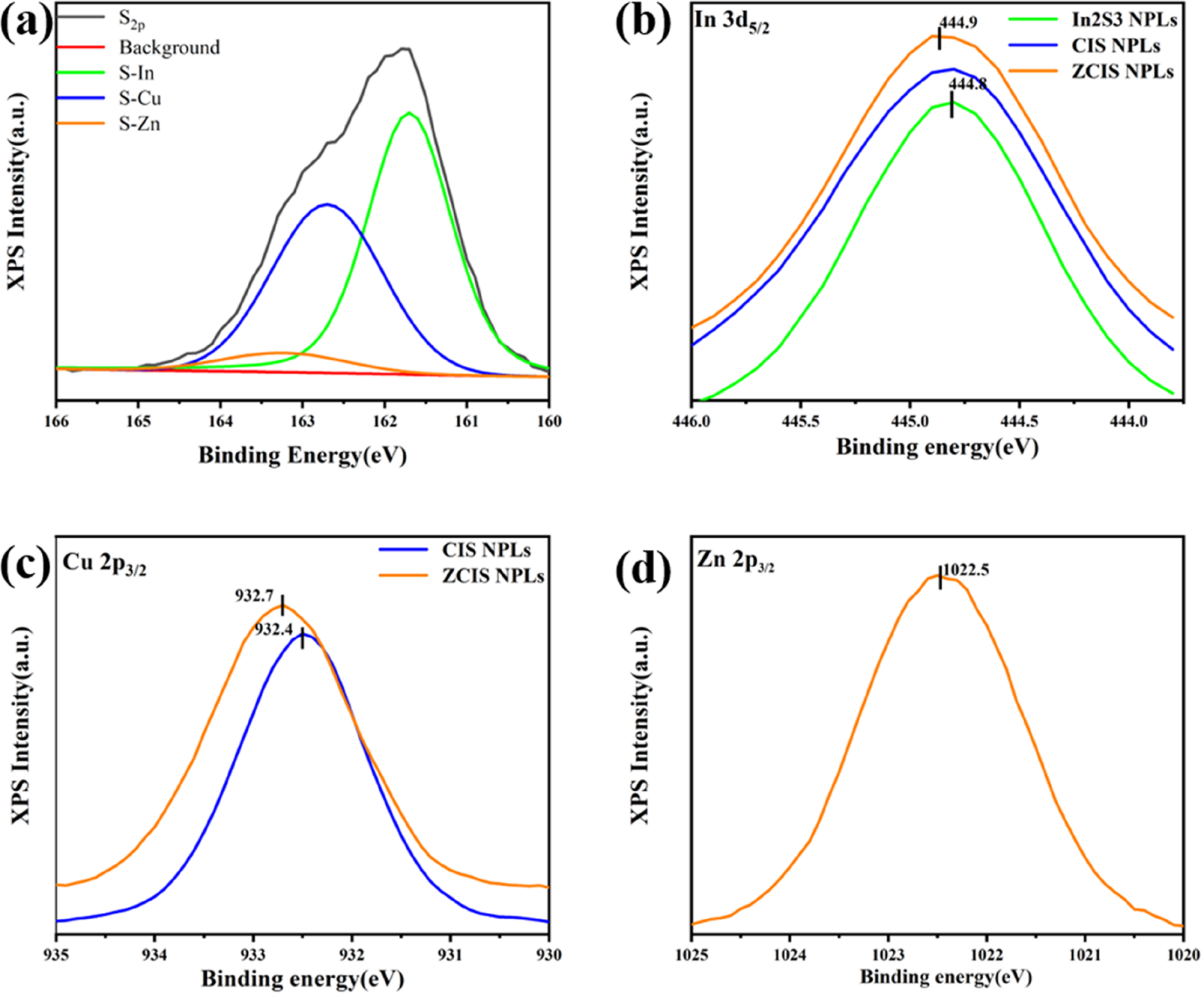
XPS spectra of In_2_S_3_, CIS, and ZCIS NPls in the region sulfur (a), indium (b), copper (c), and zinc (d) levels.

Incorporating Zn ions into a CIS matrix of colloidal QDs usually involves a combined scenario of formation of a Zn-deficient Cu–In–S core capped with a gradient Zn-enriched Cu–In–S shell.^33,38,40,41^ Such a gradient core–shell structure allows eliminating surface traps and strain at the core/shell interface. While the thickness of our ZCIS NPls is within the diameter of ZCIS QDs studied earlier where the presence of a core–gradient shell structure was verified by XPS, the 2D nature of our ZCIS NPls could not, in principle, favor such a complex structure due to almost zero surface curvature (the strong surface curvature in small QDs better suits to compensate the interfacial strain). Figure 9b shows that the introduction of Cu^+^ and Zn^2+^ ions has no noticeable effect on the XPS spectrum of In^3+^. However, after introducing Zn^2+^, the characteristic peak of Cu^+^ shifted to the higher energies but still related to Cu in a 1+ state. Such a shift can be assigned to a strong Cu^+^ and Zn^2+^ interaction inside the nanocrystal core. The characteristic peak of Zn^2+^ (Figure 9d) shifted to 4 eV to the higher energies as compared to pure ZnS, indicating that the incorporation of Zn^2+^ into CIS NPls resulted in the formation of a quaternary ZCIS phase rather than a pure ZnS shell. The successful convolution of the S characteristic band in Figure 9a into three components related to S–In, S–Cu, and S–Zn, while the S–Zn component is the weakest among two other also confirms that a large amount of Zn was introduced into the CIS core. In addition, the elemental ratio of Zn/Cu/In/S = 3.78:8.04:9.21:17.82 extracted from the XPS survey spectrum (Figure S8 in the Supporting Information) is close to the STEM-EDS data.

## Experimental Section

Chemicals indium chloride tetrahydrate (InCl_3_·4H_2_O, 98%), sulfur (S, 99.999%), zinc oxide (ZnO, 99.99%), copper iodide (CuI, 99.999%), 1-octadecene (1-ODE, 90%), 2-ethylhexanoic acid (GC, >99.0%), 1-dodecanethiol (1-DDT, 98%), and oleylamine (OLA, 90%) were purchased from Aladdin. Methanol, isopropanol, and trichloroethylene were purchased from Sinopharm Chemical Reagent Co., Ltd. All chemicals were used without further purification.

### Methods

Hydrophobic Zn–Cu–In–S nanoplatelets were synthesized via a high-temperature organometallic route. Briefly, 0.17 mmol InCl_3_·4H_2_O and 0.25 mmol S in a powder were dissolved with heating in 3 mL of octadecene (ODE) separately and cooled to room temperature. Then, 3 mL of oleylamine (OLA) was added to the InCl_3_ solution, mixed with a sulfur solution in a 25 mL three-neck round-bottom flask, and degassed for 10 min at 100 °C with stirring. The reaction mixture was then heated to 105 °C, and the system was purged with argon while being heated. At 180 °C, 0.05 mmol CuI in 1 mL of 1-dodecanethiol (DDT) was injected into the reaction mixture, which was stirred at this temperature for 3 min and then cooled to 100 °C. Then, 1 mmol ZnO dissolved in 0.5 mL of 2-ethylhexanoic acid was added to the reaction mixture. The system was reheated again to 240 °C at 4 °C min^–1^ under vigorous stirring and this temperature was maintained for 10 min. The reaction was stopped by the rapid cooling of the reaction mixture below 100 °C. The NPls were separated by adding isopropanol followed by centrifugation at 4000 rpm for 5 min. The supernatant was removed, and the NPls were dissolved in trichloroethylene. This precipitation–redispersion procedure was repeated once more to remove all soluble residuals. Insoluble residuals were separated by centrifugation of the final NPl colloidal solution in trichloroethylene at 13 000 rpm for 10 min.

### Characterization

Optical absorption spectra of NPls in trichloroethylene were recorded using a Unico (type 4802) UV–vis–NIR spectrophotometer. PL emission, excitation spectra, and the PL quantum yield were measured on an FLS1000 Photoluminescence Spectrometer in a UV–vis–NIR range. The PL quantum yield of NPls was measured relative to the reference rhodamine 6G dye in ethanol at λ_exc_ = 488 nm. Time-resolved PL measurements were carried out using a Horiba FluoroMax 4 (Kyoto, Japan), equipped with a nanosecond LED (λ = 370 nm) using the time-correlated single-photon counting. For the measurements, the aliquot of the colloidal solution was transferred into a 1 × 1 cm^2^ quartz cell at the concentration, ensuring the optical density below 0.1 at λ = 370 nm. The fitting of the PL decay curves was performed by three exponential decay. XRD analysis of the NPl powder obtained by drying an aliquot of their colloidal solution in trichloroethylene at room temperature was carried out using an Empyrean Series 2 diffractometer (Cu Kα line). XPS spectra of powdered NPls were measured with Thermo Scientific Escalab 250Xi instruments. STEM-EDX elemental mapping was done on a Tecnai G2 F20 S-TWIN instrument. Transmission electron microscopy (TEM) and high-resolution TEM (HRTEM) images were recorded using a JEM-2100HR, (JEOL) instrument operating at 200 kV. Fourier transform infrared spectroscopy (FTIR) data were measured using a Nicolet 6700 Fourier transform infrared spectrometer from Thermo Corporation, Waltham. Raman spectra were recorded using a confocal Raman microscope WiTec Alpha 300 AR (Germany) equipped with a solid-state laser excitation source emitting at λ = 488 nm.

## Conclusions

This paper demonstrated that In_2_S_3_ NPls can be successfully used as nanotemplates to prepare highly luminescent ZCIS NPls through a two-step cation exchange reaction. Starting with the as-prepared highly monodispersed binary In_2_S_3_ NPls, a subsequent cation exchange was performed, where Cu^+^ ions were partially exchanged with In^3+^ ions, leading to the formation of CIS NPls. In this step, the reaction temperature of about 180 °C is essential for the formation of ternary NPls. In a third step, Zn ethylhexanoate is reacted with CIS NPls with the formation quaternary ZCIS NPls characterized by a relatively high (28%) PL quantum yield and ca. 240 ns monoexponential PL decay. XPS analysis shows that synthesized ZCIS NPls possess a core–gradient shell structure similar to those obtained earlier in ZCIS quantum dots. We believe that ZCIS nanoplatelets can be perspective materials for practical applications as luminescence light converters, fluorescent markers, or active media of photovoltaic devices. In the last case, the advantage of ZCIS NPls lies in their 2D morphology, allowing assembling them in the form of laterally oriented multilayered films with efficient electric contacts between individual NPls, NPls, and electrodes, preferential orientation of the optical dipoles toward incident light enhancing the efficiency of the light absorption, and the absence of heavy metals in their composition.

## Supporting Information Available

Lateral size distribution of In_2_S_3_ NPls formed at different reaction temperatures (Figure S1), lateral size and thickness distribution of In_2_S_3_ NPls before injection of the Cu precursor (Figure S2), lateral size and thickness distribution of CIS NPls formed at 180 °C (Figure S3), lateral size distribution and thickness distribution of ZCIS NPls (Figure S4), resonance Raman spectra (λ_exc_ = 488 nm) of CIS and ZCIS NPls (Figure S5), the FTIR spectrum of ZCIS NPls (Figure S6), data related to the stability of ZCIS (Figure S7), and XPS survey patterns of ZCIS NPls (Figure S8) (PDF)
