# Supplementary material for: Synthesis and Optical Properties of In2S3-Hosted Colloidal Zn–Cu–In–S Nanoplatelets
Source: ACS Omega. 2021 Jul 16;6(29):18939–47. doi: 10.1021/acsomega.1c02180 (PMC8320147; doi:10.1021/acsomega.1c02180)
Supplement: Supplementary file 1 — ao1c02180_si_001.pdf [file ao1c02180_si_001.pdf]

Supporting Information

Synthesis and Optical Properties of In_2S_3-Hosted Colloidal Zn−Cu−In−S Nanoplatelets

Ze Yuan[a], Lanlan Yang[a], Dongni Han[a], Guorong Sun[a], Chenyu Zhu[a], Yao Wang[a], [a] Qiao Wang[c], Mikhail Artemyev [b*], Jianguo Tang [a*]

[a] Institute of Hybrid Materials, National Center of International Joint Research for Hybrid Materials Technology, National Base of International Sci. & Technology Cooperation on Hybrid Materials, Qingdao University, 308 Ningxia Road, Qingdao 266071, People's Republic of China

[b] Research Institute for Physical Chemical Problems of the Belarusian State University, Minsk 220006, Belarus

* Corresponding author, e-mail: tang@qdu.edu.cn

m_artemyev@yahoo.com

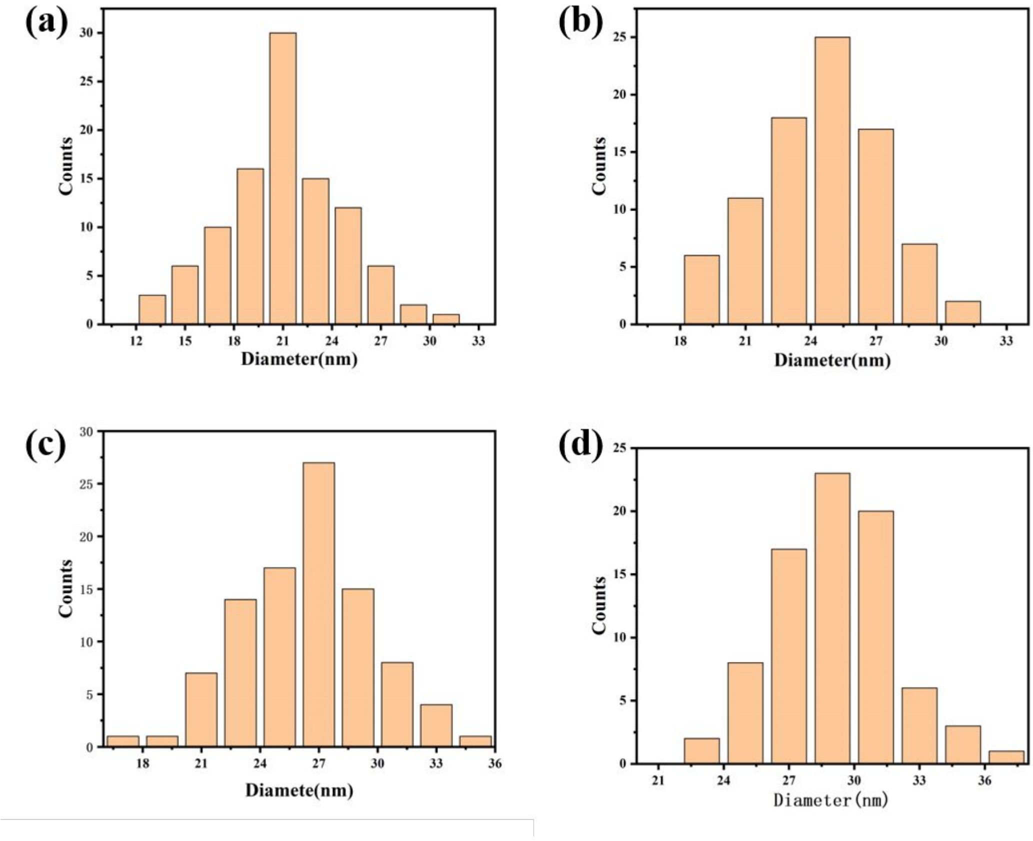

Figure S1. Lateral size distribution of In_2S_3 NPLs formed at different reaction temperature: 160 ℃ (a), 180 ℃ (b), 200 ℃ (c), 220 ℃ (d).

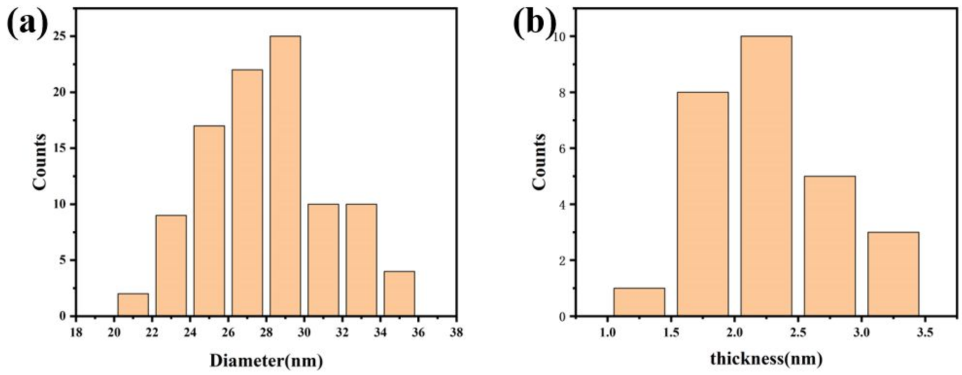

Figure S2. Lateral size and thickness distribution of In_2S_3 NPls before injection of Cu precursor.(a) Lateral size (b)thickness

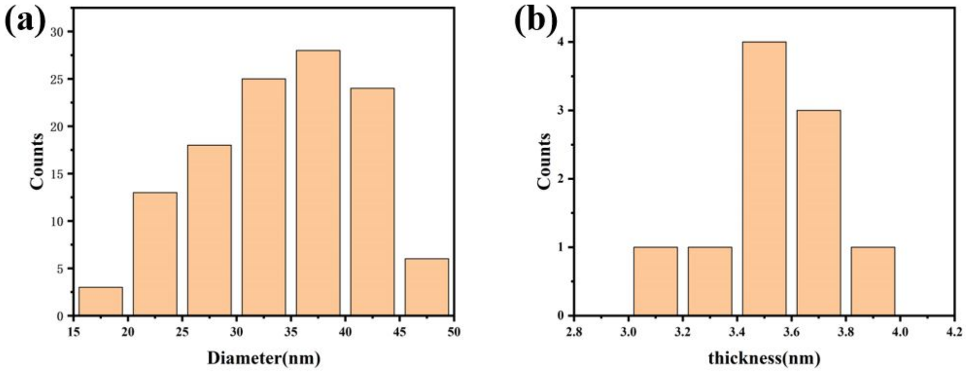

Figure S3. Lateral size and thickness distribution of CIS NPls formed at 180 °C. (a) Lateral size (b)thickness

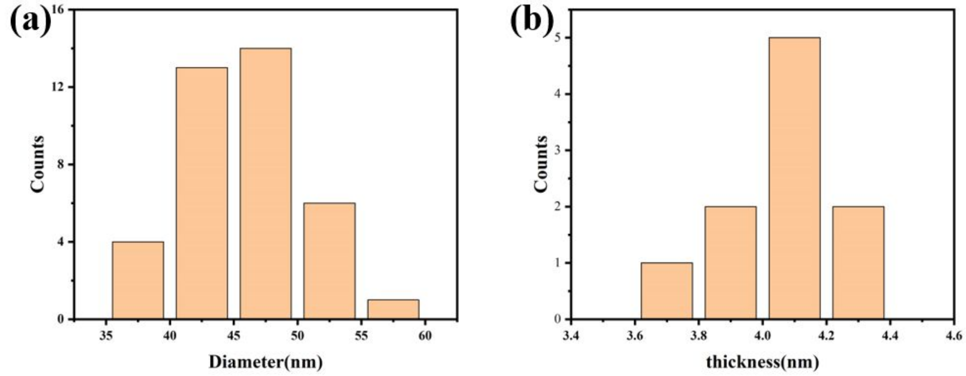

Figure S4. Lateral size distribution and thickness distribution of ZCIS NPLS. (a) Lateral size (b)thickness

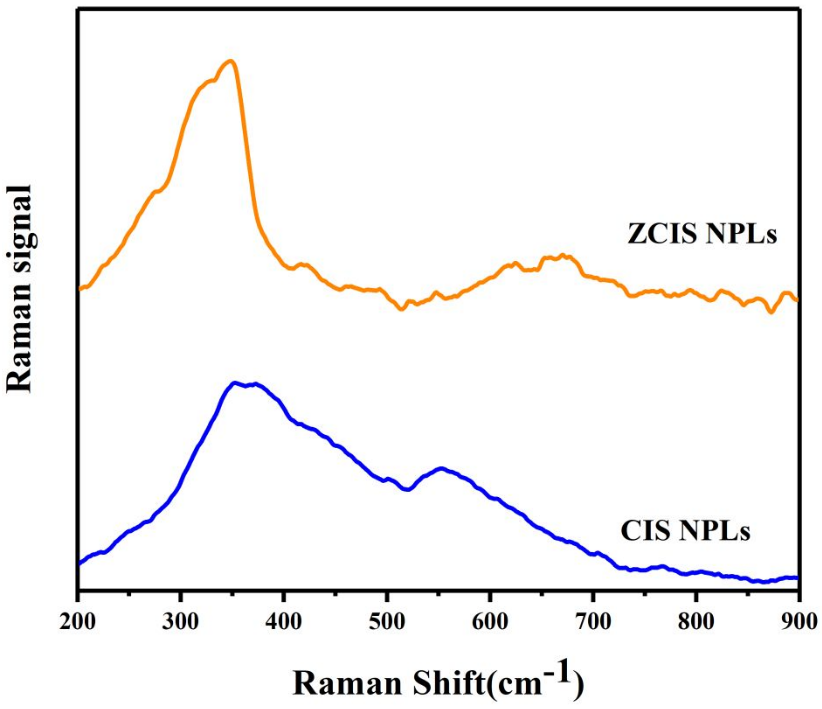

Figure S5. Resonance Raman spectra (λ_{exc} = 488 nm) of CIS, and ZCIS NPLs.

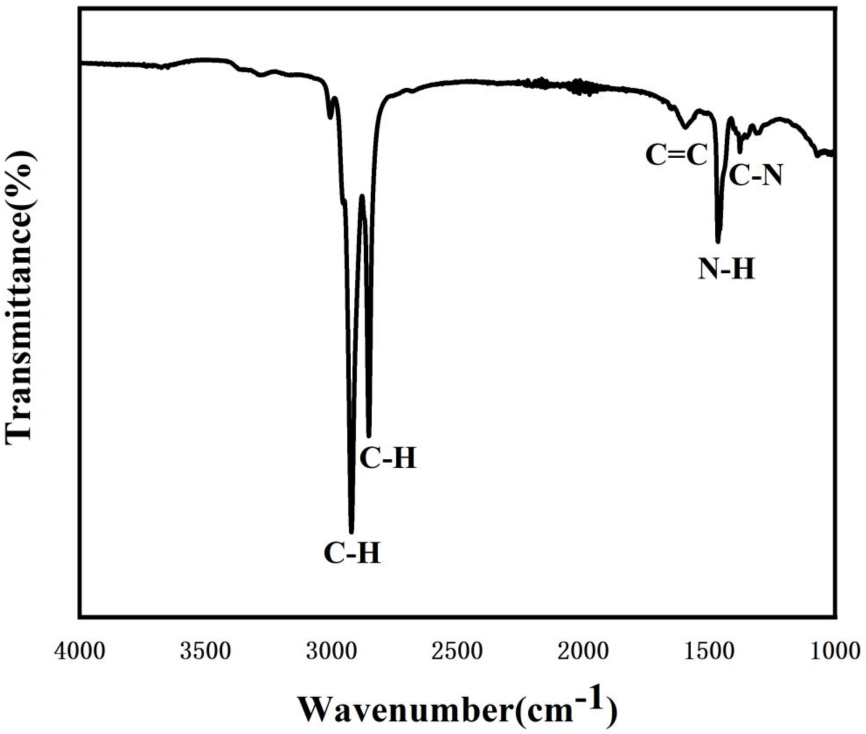

Figure S6. FTIR spectrum of ZCIS NPLS

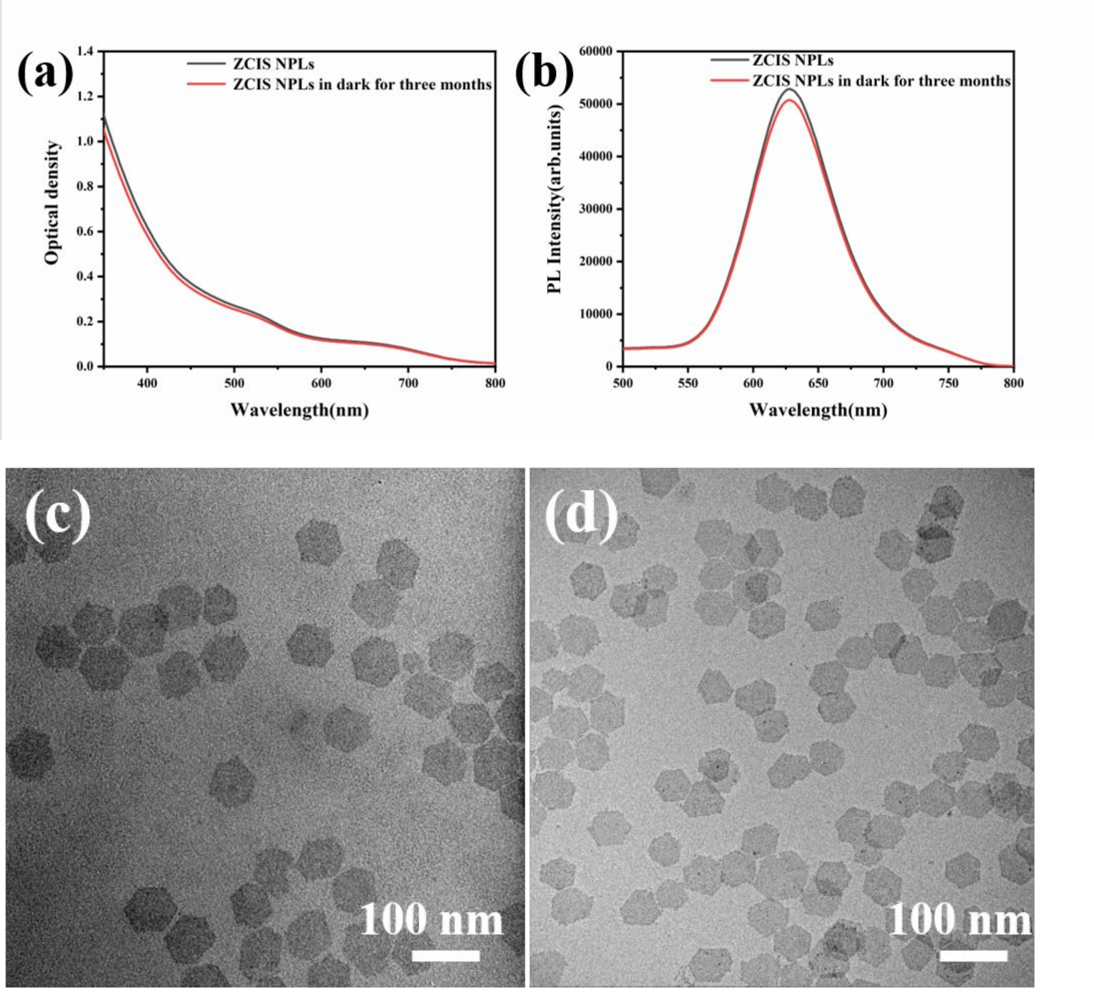

Figure S7. Absorption (a) and PL (b) spectra of ZCIS NPLs before and after long-term storage.

TEM images of as-synthesized (c) and long-term stored (d) ZCIS NPLS.

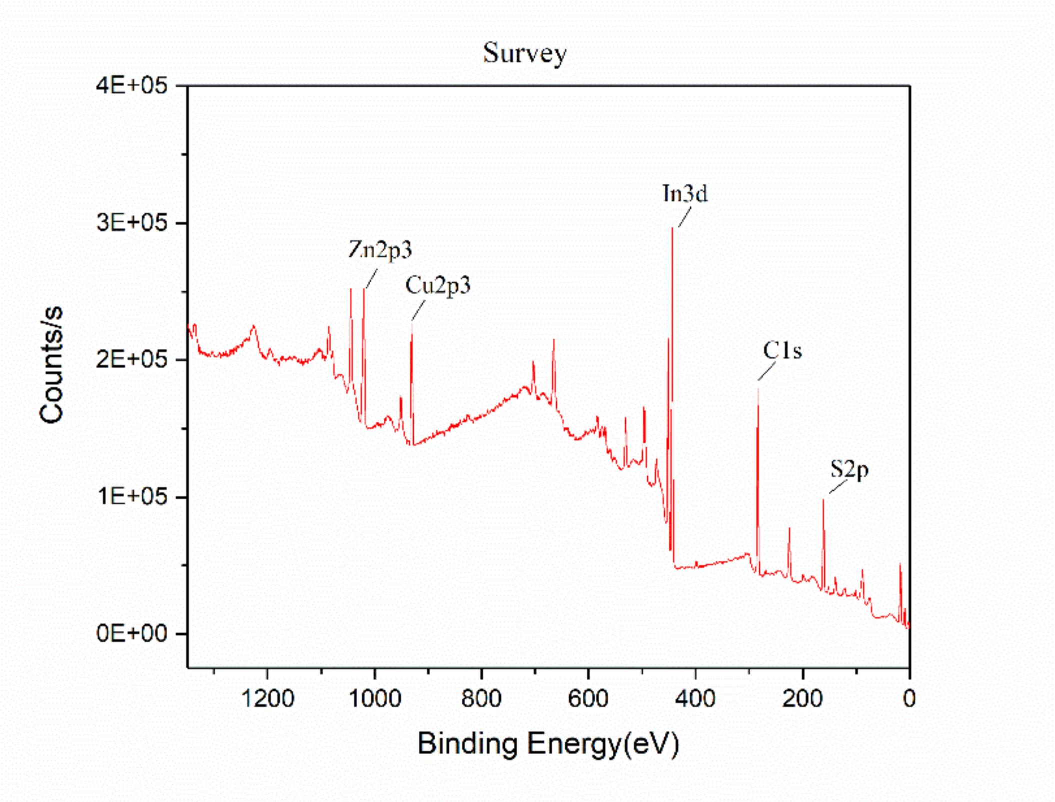

Figure S8. XPS Survey patterns of ZCIS NPLs.
